# Georgia Pharmaceutical Association

**Published:** 1878-03

**Authors:** 


GEORGIA PHARMACEUTICAL ASSOCIATION.—The third annual meeting of this body will be held in Augusta, on the second Tuesday, 9th day, of April, proximo.

The organization of this association was effected in Macon, on October 20th, 1875, in pursuance of a call by Dr. Fred King, of Atlanta, and Mr. S. W. Hunt, of Macon; and the first annual meeting was held at the same place on the 11th of April, 1876. We learn that important matters will be considered at the approaching meeting in Augusta, and that a full attendance is, therefore, desirable.

